# Gaps in Prenatal Hepatitis B Screening and Management of HBsAg Positive Pregnant Persons in the U.S., 2015–2020

**DOI:** 10.1016/j.amepre.2023.01.041

**Published:** 2023-03-10

**Authors:** Thi T. Hang Pham, Nimisha Maria, Vivian Cheng, Brandon Nguyen, Mehlika Toy, David Hutton, Erin E. Conners, Noele P. Nelson, Joshua A. Salomon, Samuel So

**Affiliations:** 1 Asian Liver Center, Department of Surgery, Stanford University School of Medicine, Palo Alto, California; 2 Department of Health Management and Policy, School of Public Health, University of Michigan, Ann Arbor, Michigan; 3 Division of Viral Hepatitis, Centers for Disease Control and Prevention, Atlanta, Georgia; 4 Center for Health Policy/Center for Primary Care and Outcomes Research, Stanford University, Stanford, California

## Abstract

**Background:**

The Advisory Committee for Immunization Practices (ACIP) recommends testing all pregnant women for hepatitis B surface antigen (HBsAg) and testing HBsAg–positive pregnant women for hepatitis B virus deoxyribonucleic acid (HBV DNA). HBsAg–positive pregnant persons are recommended by the American Association for the Study of Liver Diseases to receive regular monitoring, including alanine transaminase (ALT) and HBV DNA and antiviral therapy for active hepatitis and to prevent perinatal HBV transmission if HBV DNA level is >200,000 IU/mL.

**Methods:**

Using Optum Clinformatics Data Mart Database claims data, pregnant women who received HBsAg testing and HBsAg–positive pregnant persons who received HBV DNA and alt testing and antiviral therapy during pregnancy and after delivery during January 1, 2015–December 31, 2020 were analyzed.

**Results:**

Among 506,794 pregnancies, 14.6% did not receive HBsAg testing. Pregnant women more likely to receive testing for HBsAg (*p*<0.01) were persons aged ≥20 years, were Asian, had >1 child, or received education beyond high school. Among the 0.28% (1,437) pregnant women who tested positive for hepatitis B surface antigen, 46% were Asian. The proportion of HBsAg–positive pregnant women who received HBV DNA testing during pregnancy and in the 12 months after delivery was 44.3% and 28.6%, respectively; the proportion that received hepatitis B e antigen was 31.6% and 12.7%, respectively; the proportion that received ALT testing was 67.4% and 47%, respectively; and the proportion that received HBV antiviral therapy was 7% and 6.2%, respectively.

**Conclusions:**

This study suggests that as many as half a million (~14%) pregnant persons who gave birth each year were not tested for HBsAg to prevent perinatal transmission. More than 50% of HBsAg–positive persons did not receive the recommended HBV–directed monitoring tests during pregnancy and after delivery.

## INTRODUCTION

In the U.S., there are an estimated 860,000–2.4 million people living with chronic hepatitis B (CHB) who are at risk of premature death from liver cirrhosis and hepatocellular carcinoma if they are not monitored for disease progression and receive antiviral therapy when indicated.^1,2^ The Centers for Disease Control and Prevention (CDC) estimated that 20,678 women who gave birth in 2015 were infected with hepatitis B virus (HBV).^3^ Pregnant women infected with HBV can transmit the infection to their newborns. Without immunoprophylaxis, as many as 90% of infants born to hepatitis B surface antigen (HBsAg)– and hepatitis B e antigen (HBeAg)–positive mothers (who are generally highly viremic) and 5%–20% of infants born to HBsAg-positive and HBeAg-negative mothers will develop CHB.^4^ Infants who develop CHB are at the highest risk of death from liver cirrhosis and hepatocellular carcinoma later in life.^2^ By CDC estimates, about 950 infants each year became chronically infected with HBV from perinatal transmission in the U.S.^5^

The Advisory Committee on Immunization Practices and U.S. Preventive Services Task Force recommend universal prenatal HBsAg testing at each pregnancy to identify women infected with HBV.^2,6^ In 2018, Advisory Committee on Immunization Practices also recommended that HBsAg-positive pregnant women receive testing for HBV deoxyribonucleic acid (DNA) to identify women with high viral load. Twenty-six states have laws that mandate prenatal hepatitis B testing.^7^ On the basis of claims data, CDC reported that between 2011 and 2014, 18% of commercially insured pregnant women were not tested for HBsAg and in that 2014, 12.3% of commercially insured and 16.4% of Medicaid-enrolled women who gave birth did not receive prenatal HBsAg testing.^8,9^

Patients with CHB require chronic disease management, including regular monitoring for active hepatitis and progression to liver cirrhosis. Hepatitis flares with elevated alanine transaminase (ALT) and liver injury can occur during pregnancy and after delivery and may require antiviral therapy.^10,11^ The American Association for the Study of Liver Disease in 2007 recommended that HBsAg-positive persons, including pregnant women, receive CHB disease evaluation and monitoring, including testing for ALT, HBeAg, and HBV DNA, and HBV antiviral therapy for those with active hepatitis.^12^ In 2016, the American Association for the Study of Liver Disease further recommended antiviral therapy to prevent perinatal HBV transmission if HBV DNA is over 200,000 IU/mL.^12,13^ Harris et al.^8^ found that among the pregnant women between January 1, 2011 and June 30, 2014, only 42% of the HBsAg-positive women during pregnancy and 39% after delivery received claims for HBV-directed monitoring tests defined as ALT and HBV DNA or HBeAg^8^; 13% received claims for antiviral therapy during pregnancy but only 1.6% after delivery.

The goal of this study is to assess whether since 2014, there has been an increase in HBsAg testing among pregnant persons with commercial insurance on the basis of claims data and the proportion of HBsAg-positive persons who received HBV DNA, HBeAg, ALT testing, and antiviral therapy during pregnancy and in the first 12 months after delivery. Multiple factors were analyzed to identify gaps and disparities to improve HBsAg screening and CHB management among pregnant persons.

## METHODS

### Study Sample

Deidentified data of patients’ demographics, diagnosis, and insurance claims were obtained from the Optum Clinformatics Data Mart (Optum 5.0) database between January 1, 2015 and December 31, 2020 hosted at the Stanford Center for Population Health Sciences. In 2014, approximately 19% of the U.S. population in commercial health plans and 19% of those in Medicare Advantage plans were represented in Optum’s administrative data assets.^14^ Unique pregnancies were identified by searching medical claims for live birth and delivery-related diagnosis and procedure codes (Appendix Tables 1 and 2, available online). Pregnancies among persons aged 15–55 years who had at least 1 delivery or live birth-related code entered between January 01, 2015 and December 31, 2019 and were in continuous enrollment at least 42 weeks before the delivery date were included in this study (Appendix Figure 1, available online).

### Measures

To evaluate HBsAg testing during pregnancy, medical claims were searched for current procedural terminology codes for laboratory tests that included HBsAg within 42 weeks before delivery (Appendix Table 3, available online). *Unique pregnancies with CHB diagnosis* were defined as having at least one International Classification of Diseases, Ninth Revision or ICD-10 diagnosis code for CHB (Appendix Table 4, available online). Pregnancies in which the mothers had at least 1 diagnosis code for HIV or hepatitis C virus before delivery were excluded from the monitoring and treatment evaluation. CHB monitoring and treatment during pregnancy and after delivery was based on unique HBsAg-positive pregnancies with at least 1 current procedural terminology code for laboratory tests for ALT, HBV DNA, and HBeAg (Appendix Table 5, available online) and prescription claims for at least 1 brand or generic HBV antiviral medication (Appendix Table 6, available online) within 42 weeks before the delivery date and within 12 months after delivery. This secondary analysis of deidentified insurance claims data was approved under the Stanford University Center for Population Health Sciences umbrella IRB 40974.

### Statistical Analysis

The Stata 12.0 statistical software was used for data analysis. Descriptive statistical analysis was performed to describe the demographics of the unique pregnancies and to calculate the key outcome measurements (HBsAg testing, HBV DNA, ALT, and HBeAg monitoring and antiviral treatment). Univariable and multivariable logistic regression with the generalized estimation equation model taking into consideration persons who had more than 1 pregnancy in the data set were used to evaluate the correlation between demographic characteristics of pregnancies with HBV testing, disease monitoring, and antiviral treatment. Variables with *p*<0.25 in univariable analysis were included in multivariable analysis. Adjusted ORs and their 95% CIs were used to provide further insight regarding the relative importance of each independent variable on the outcome variable. Degree of statistical significance was declared at a *p*≤0.05.

## RESULTS

A total of 625,689 unique pregnancies (565,858 unique pregnant persons) were enrolled in the Optum database between January 1, 2015 and December 31, 2019 (Appendix Figure 1, available online). Among the 506,794 pregnancies (459,125 unique pregnant persons) in continuous enrollment at least 42 weeks before delivery, the median age was 31 years; 93.8% were aged 20–39 years, 65.9% were White, 14.6% were Hispanic, 9.3% were Black, and 8.4% were Asian. Most of them (88.8%) had at least one child before their pregnancies between 2015 and 2019 (Appendix Figure 1, available online, Appendix Table 7, available online).

**Figure 1.**
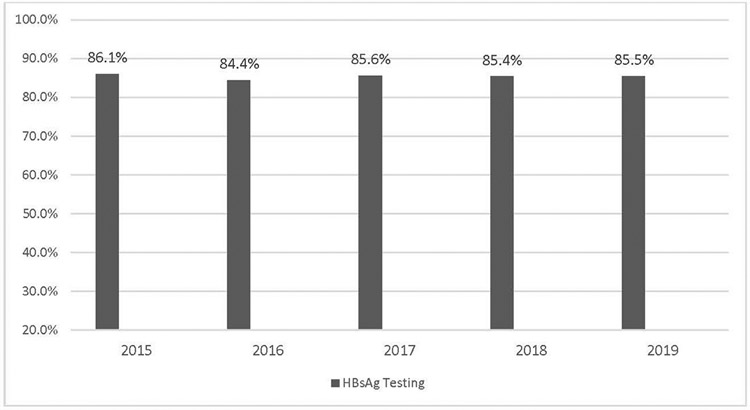
Percentage of pregnancies with HBsAg testing by year, 2015–2019. HBsAg, hepatitis B surface antigen.

Of 506,794 pregnancies that were in continuous enrollment at least 42 weeks before delivery, there were 432,607 (85.4%) who had at least one claim for HBsAg test during pregnancy (Appendix Figure 1, available online). The percentage of pregnancies that had HBsAg testing each year ranged from 84.4% to 86.1% during the study years 2015–2019 (Figure 1).

The prenatal testing rate for HBsAg among pregnant persons varied by age group, ethnicity, education level, and number of children. Multivariable regression analysis showed that pregnant persons aged ≥20 years, with an educational level beyond a high-school diploma, who were Asian, and who had one or more children were significantly more likely (*p*<0.01) to receive HBsAg testing (Table 1).

There were 1,437 of 506,794 (0.28%) unique pregnancies (1,309 unique persons) with at least 1 CHB diagnosis before delivery. The majority (98.8%) of the HBsAg-positive pregnancies were aged ≥20 years, and 92.8% had >1 child (Appendix Table 8, available online). Among the HBsAg-positive pregnant women, 46.0% were Asian, 29.1% were White, 12.9% were Black, and 9.1% were Hispanic; 35.1% had a bachelor’s or postgraduate degree; and 47.3% had postsecondary education but less than bachelor’s degree.

**Figure 2.**
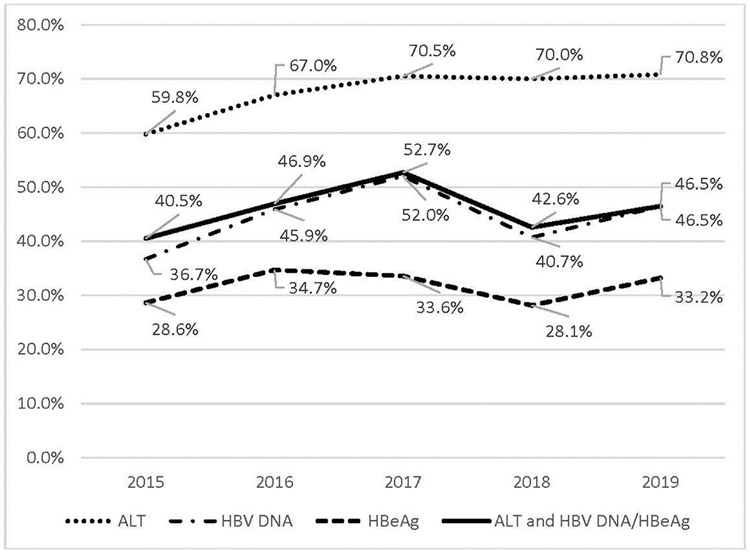
Percentage of CHB pregnancies with recommended follow-up laboratory testing, by test type, 2015–2019. ALT, alanine transaminase; CHB, chronic hepatitis B; HBeAg, hepatitis B e antigen; HBV DNA, hepatitis B virus deoxyribonucleic acid.

Among the 1,437 unique pregnancies with CHB diagnosis, 637 (44.3%) had claims for ≥1 HBV DNA (27.4% had claims for both HBV DNA and HBeAg, 17% had claims for HBV DNA only), and 4.3% had claims for HBeAg only during pregnancy. The proportion with claims for ≥1 HBV DNA test during pregnancy increased from 36.7% to 52% between 2015 and 2017 (*p*<0.01) but dropped back to 40.7% in 2018 and 46.5% in 2019 (Figure 2 and Appendix Table 9, available online). A total of 969 (67.4%) pregnancies with CHB had claims for ≥1 ALT test. The proportion with claims for ≥1 ALT test during pregnancy increased from 59.7% to 70.4% (*p*<0.01) between 2015 and 2017 but remained at 70% in 2018 and 2019 (Figure 2 and Appendix Table 9, available online).

There were 659 pregnant women (45.8%) who received *HBV-directed monitoring*, defined as having at least one ALT test plus one HBV DNA or HBeAg test during pregnancy. Asian persons had the highest HBV-directed monitoring rate during pregnancy at 60.3% than 49.5% for Black, 31.8% for Hispanic, and 24.4% for White persons (Table 2). Multivariable analysis showed that White and Hispanic persons had significantly lower (OR=0.21, 95% CI=0.16, 0.28 and OR=0.31, 95% CI=0.20, 0.47, respectively, *p*<0.01) HBV-directed monitoring during pregnancy than Asian persons. Persons with an educational level beyond high school or who had one or two children also have higher HBV-directed monitoring during pregnancy (Table 2).

The proportion of pregnancies receiving HBV-directed monitoring within the 12 months after delivery was lower than that during pregnancy at 26.1%. Only 47% had claims for ALT, and 28.6% had claims for HBV DNA within the 12 months after delivery (Appendix Figure 2, available online). Multivariable analysis found that persons who were significantly more likely to receive testing for HBV-directed monitoring within the 12 months after delivery were Asian than White and Hispanic (*p*<0.01) and persons with at least two children (*p*<0.05) (Appendix Table 10, available online).

Among the 1,437 unique pregnancies with CHB diagnosis codes, 101 pregnancies (7%) had pharmacy claims for HBV antiviral during pregnancy (Appendix Figure 1, available online). A significantly higher proportion of Asian HBsAg-positive pregnant persons (10.6%, *p*<0.01) had claims for HBV antiviral therapy during pregnancy than Black (3.2%), Hispanic (2.3%), and White persons (4.3%) (Appendix Table 11, available online). There were no differences in pregnant persons who received antiviral treatment by age group, education level, and number of children. Among the cohort of 962 pregnant women who were in continuous enrollment at least 12 months after delivery, 60 (6.2%) received claims for antiviral treatment in the 12 months after delivery. Asians were more likely to have claims for antiviral therapy during pregnancy and after delivery (Appendix Table 12, available online).

## DISCUSSION

In this study of persons aged 15–50 years with commercial insurance enrolled in the Optum database who gave birth between January 1, 2015 and December 31, 2019, about 15% of the pregnancies did not receive a hepatitis B test. Among the pregnancies who received testing between 2015 and 2019, the prevalence of HBsAg was 0.28%. Asian persons accounted for almost half of HBsAg-positive pregnancies, although Asian persons only comprised 8.4% of the pregnancies between 2015 and 2019. The disproportionately high proportion of Asian persons who were HBsAg positive is consistent with the high prevalence of CHB in the Asian community. Consistent with the high national and global hepatitis B vaccination coverage in children and adolescents, persons aged <20 years accounted for only 0.2% of HBsAg-positive pregnancies.

Elimination of perinatal hepatitis B is a pillar of the U.S. national strategy to eliminate hepatitis B by 2030.^15^ In this study, the proportion of pregnant women who were not tested for HBsAg each year (~15%) has not declined between 2015 and 2019. Compared with the CDC reports that 12.3%–18% of pregnant women on commercial insurance in 2011–2014 and 16.4% on Medicaid in 2014 were not tested for hepatitis B, this study suggests that there has likely been little or no improvement to close the gap in prenatal hepatitis B testing in the U.S. in the last decade.^8,9^ With an estimated 3.65 million births annually and 0.28% HBsAg prevalence among pregnant persons in the U.S., if the prenatal HBsAg testing rate remains at 85.5%, it would suggest that as many as half a million pregnancies each year would not receive HBsAg testing, resulting in a failure to identify as many as 1,480 HBsAg-positive pregnant persons at risk for perinatal transmission each year.^16^

To address the stalled screening rates, several recommendations should be considered. Health plans and providers’ professional organizations, including ACOG (American College of Obstetricians and Gynecologists), should also recommend that prenatal care providers use obstetrics/prenatal panels that included the HBsAg test. CDC should work with the electronic health records industry to build clinical decision support tools for prenatal HBsAg testing and reflex testing for HBV DNA, HBeAg, and ALT for HBsAg-positive persons to improve HBV-directed testing during pregnancy and after delivery. CDC should recommend the National Committee for Quality Assurance to adopt a measure to monitor compliance with prenatal HBsAg testing and include the new measure in the Healthcare Effectiveness Data and Information Set, which is used to compare the performance and quality of health plans. States should mandate prenatal hepatitis B testing (beyond the current 26 states) as part of the national strategy to improve the national screening rate.

Testing for HBV DNA in HBsAg-positive pregnancies would identify persons with high viral load who are recommended to receive antiviral therapy to prevent perinatal transmission.^2,13,14^ In this study, only 44.3% of HBsAg-positive pregnancies had claims for HBV DNA testing. HBV DNA testing increased from 35.5% in 2015 to 51.9% in 2017 but dropped back to 40.7% in 2018 and 46.5% in 2019 (Figure 2 and Appendix Table 9, available online).

Monitoring of ALT during pregnancy and after delivery is important to detect ALT flares that may require antiviral therapy. In this study, about 30% of the HBsAg-positive pregnancies did not receive ALT testing. ALT testing during pregnancy increased from 59.7% in 2015 to 70.4% in 2017 but remained at 70% in 2018 and 2019. In a multicenter retrospective analysis of two community gastroenterology clinics and two tertiary medical centers in the U.S., 6% of women during pregnancy and 10% of women within the first three months after delivery developed ALT flares.^17^ All the ALT flares during pregnancy were reported as severe with ALT >10 × upper limit of normal, including one woman who developed hepatic decompensation at 33 weeks of gestation. Among the women with ALT flare during pregnancy, 50% required antiviral therapy.

In this study, HBsAg-positive persons who were less likely to have received testing for ALT and HBV DNA or HBeAg during pregnancy were White and Hispanic, were first-time mothers, or had less than a 12th-grade education.

CHB is a chronic liver disease that requires long-term monitoring to assess disease activity and the need for antiviral therapy.^12-14^ In this study, more than 50% of the pregnant persons with CHB diagnosis did not receive the recommended monitoring tests after pregnancy. In 2019, only 46.6% received testing for ALT, 25.8% received testing for HBV DNA, and 11.7% received testing for HBeAg within 12 months after delivery (Appendix Figure 2, available online and Appendix Table 9, available online).

Strategies to increase HBV-directed testing and management during pregnancy should include increased efforts to provide all the perinatal providers (including doctors and nurses) with an easy-to-follow algorithm for testing and referral for HBV-directed care. The publication of the CDC/ACOG Screening and Referral Algorithm for hepatitis B Among Pregnant Women in 2015 may have contributed to the increased ALT and HBV DNA testing rate between 2015 and 2017.^18^ Prenatal care providers should also provide their HBsAg-positive pregnant persons culturally and linguistically appropriate educational information about CHB facts, prevention, monitoring, and treatment. This is particularly pertinent because some of them may only become aware of their infection through prenatal testing and are not aware of the risks of CHB and the benefits of disease monitoring and treatment. Chao and colleagues^16^ found in a survey of 138 practicing obstetricians in a CHB high-prevalence county in California, that only 60.9% routinely advised HBsAg-positive pregnant women to seek specialist evaluation for monitoring and antiviral treatment and that only 48.6% routinely provided them with CHB information. Expanding the role of the CDC-funded Perinatal hepatitis B Prevention Program would also help to ensure that HBsAg-positive pregnant persons receive the recommended testing and management.^19^

### Limitations

There were several limitations in the study. The Optum database is not representative of the entire U.S. population because it does not include women who are uninsured or covered by Medicaid or Veterans Administration and therefore may not be representative of the status of hepatitis B testing and management of HBsAg-positive pregnant women in the U.S. The proportion of pregnant women who did not receive HBsAg testing in this study (14.6%) is nevertheless within the range of 12.3%–18% reported among pregnancies between 2011 and 2014.^8,9^ Another limitation is that claims data in this study does not provide information about healthcare providers and clinical parameters, including HBsAg-positive women on antiviral therapy before they became pregnant, women who stopped antiviral treatment when they became pregnant, and whether antiviral therapy was prescribed for hepatitis flares during pregnancy or to prevent perinatal transmission. Likewise, the data do not provide information to determine whether women who did not receive ALT and HBV DNA testing or antiviral treatment were due to noncompliance or because they were not ordered or prescribed by the healthcare providers.

## CONCLUSIONS

This study suggests that as many as half a million pregnant persons who gave birth in the U.S. each year were not tested for hepatitis B and that this could have resulted in a failure to identify as many as 1,480 HBsAg-positive pregnant persons at risk for perinatal transmission of hepatitis B each year. The study further found that less than half of the HBsAg-positive pregnant persons received HBV-directed monitoring during pregnancy. These gaps in screening and monitoring may have contributed to the estimated 950 infants who develop CHB each year in the U.S. A national strategy to eliminate mother-to-child transmission of hepatitis B by 2030 would need a call to action to strengthen the implementation of the existing recommendations and to introduce new healthcare providers, pregnant persons, and health systems–related strategies to improve prenatal hepatitis B screening, HBV-directed monitoring, and management of HBsAg-positive pregnant persons.

## Supplementary Material

Appendix

## Figures and Tables

**Table 1.** Demographic Factors Associated With HBsAg Testing During Pregnancy Among 506,794 Unique Pregnancies, 2015–2019

Characteristics	Tested (%)	Univariate analysis		Multivariate analysis	
		OR (95% CI)	*p*	AOR (95% CI)	*p*
Age at the time of pregnancy, years					
15–19	5,122 (78.6)	(ref)			
20–29	142,209 (83.9)	1.42 (1.33–1.50)	**<0.01** **	1.24 (1.17–1.32)	**<0.01** **
30–39	263,168 (86.4)	1.73 (1.63–1.84)	**<0.01** **	1.37 (1.29–1.46)	**<0.01** **
40–55	22,108 (84.9)	1.54 (1.43–1.64)	**<0.01** **	1.22 (1.14–1.31)	**<0.01** **
Race/ethnicity					
Asian	37,407 (87.8)	(ref)			
Black	39,605 (83.7)	0.73 (0.70–0.76)	**<0.01** **	0.81 (0.77–0.84)	**<0.01** **
Hispanic	61,883 (84.2)	0.74 (0.72–0.77)	**<0.01** **	0.81 (0.78–0.84)	**<0.01** **
White	284,777 (85.5)	0.82 (0.79–0.84)	**<0.01** **	0.85 (0.83–0.88)	**<0.01** **
Education					
<12th grade	1,640 (82.3)	(ref)			
High-school diploma	77,750 (83.6)	1.09 (0.97–1.23)	0.13	1.07 (0.95–1.20)	0.29
Less than bachelor’s	232,736 (85.5)	1.27 (1.12–1.41)	**<0.01** **	1.16 (1.03–1.30)	**0.02** *
Bachelor’s degree plus	118,693 (86.4)	1.36 (1.12–1.53)	**<0.01** **	1.16 (1.03–1.31)	**0.01** *
Number of children					
0	44,932 (79.7)	(ref)			
1	140,836 (85.2)	1.46 (1.42–1.50)	**<0.01** **	1.35 (1.31–1.38)	**<0.01** **
2	150,768 (86.6)	1.64 (1.60–1.68)	**<0.01** **	1.47 (1.44–1.51)	**<0.01** **
≥3	95,097 (85.5)	1.62 (1.58–1.67)	**<0.01** **	1.47 (1.43–1.51)	**<0.01** **

*Note:* Boldface indicates statistical significance (*p<0.05and **p<0.01).

HBsAg, hepatitis B surface antigen

**Table 2.** Demographics Factors Associated With HBV-Directed Monitoring (ALT + HBV DNA/HBeAg) Among Pregnancies With CHB Diagnosis, 2015–2019

Characteristics	Number (%)	Univariate analysis		Multivariate analysis	
		OR (95% CI)	*p*	AOR (95% CI)	*p*
Age, years					
15–29	108 (46.0)				
30–39	474 (46.2)	1.01 (0.76–1.34)	0.95	0.79 (0.58–1.09)	0.15
40–55	77 (43.8)	0.9 (0.62–1.35)	0.66	0.68 (0.44–1.05)	0.08
Race/ethnicity					
Asian	399 (60.3)				
Black	91 (49.5)	0.65 (0.47–0.90)	**0.01** *	0.73 (0.51–1.04)	0.08
Hispanic	41 (31.8)	0.30 (0.20–0.45)	**<0.01** **	0.31 (0.20–0.47)	**<0.01** **
White	102 (24.4)	0.21 (0.16–0.28)	**<0.01** **	0.21 (0.16–0.28)	**<0.01** **
Education					
High-school completion or less	90 (37.19)				
Less than bachelor’s	324 (47.7)	1.53 (1.14–2.07)	**<0.01** **	1.57 (1.13–2.16)	**<0.01** **
Bachelor’s degree plus	242 (47.9)	1.55 (1.14–2.12)	**<0.01** **	1.50 (1.06–2.11)	**0.02** *
Number of children					
0	34 (32.7)				
1	242 (50.8)	2.13 (1.36–3.33)	**<0.01** **	2.16 (1.31–3.54)	**<0.01** **
2	266 (46.3)	1.78 (1.41–2.76)	**0.01** *	1.77 (1.08–2.91)	**0.02** *
≥3	117 (41.3)	1.45 (0.90–2.33)	0.12	1.51 (0.90–2.56)	0.11

*Note:* Boldface indicates statistical significance (*p<0.05a and **p<0.01).

ALT, alanine transaminase; CHB, chronic hepatitis B; HBeAg, hepatitis B e antigen; HBV, hepatitis B virus; HBV DNA, hepatitis B virus deoxyribonucleic acid.
