# Supplementary material for: Gaps in Prenatal Hepatitis B Screening and Management of HBsAg Positive Pregnant Persons in the U.S., 2015–2020
Source: Am J Prev Med. Author manuscript; Available in PMC 2024 Jul 1. (PMC10994214; doi:10.1016/j.amepre.2023.01.041)

Appendix

Gaps in prenatal hepatitis B screening and management of HBsAg positive pregnant persons in the United States, 2015-2020

Thi T. Hang Pham MD, MPH, Nimisha Maria BA, Vivian Cheng, Brandon Nguyen, Mehlika Toy PhD, David Hutton PhD, Erin E. Conners PhD, MPH , Noele P. Nelson PhD, MPH, Joshua A Salomon PhD, and Samuel So MBBS, FACS. American Journal of Preventive Medicine.

Appendix Table 1. ICD Codes for Live Birth

Appendix Table 2. CPT Codes for Delivery

Appendix Table 3. CPT Codes for Hepatitis-B Screening

Appendix Table 4. ICD 9/10 Codes for Chronic Hepatitis B

Appendix Table 5. CPT Codes for CHB Monitoring

Appendix Table 6. Antiviral Treatment Codes

Appendix Table 7. Demographics characteristics of 506,794 unique pregnancies between 1/1/2015 and 12/31/2019 in continuous enrollment at least 42 weeks before delivery

Appendix Table 8. Demographic characteristics of 1,437 HBsAg positive pregnancies, 2015-2019

Appendix Table 9. Monitoring of CHB with testing for ALT, HBV DNA and HBeAg among 1,437 unique pregnancies diagnosed with CHB, 2015-2019

Appendix Table 10. Demographics factors associated with HBV-directed monitoring (ALT + HBV DNA/HBeAg) 0-12 months after delivery among 962 unique pregnancies with CHB diagnosis in continuous enrollment at least 12 months after delivery, 2015-2020

Appendix Table 11. Correlation analysis of demographics on HBV antiviral treatment during pregnancy among 1,437 unique pregnancies, 2015-2019

Appendix Table 12. Demographics factors associated with claims for HBV antiviral treatment in the 12 months after delivery among the pregnancies with CHB diagnosis in continuous enrollment at least 12 months after delivery, 2015-2020

Appendix Figure 1. Flow chart cascade of hepatitis B testing, care, and treatment among pregnancies in Optum, 2015-2020.

Appendix Figure 2. HBV disease monitoring in the 12 months after delivery, 2015-2020

Appendix Table 1. ICD Codes for Live Birth

	ICD9/10 Codes	Code Description
Live Birth	Z370	Single live birth
	Z375	Other multiple births, all liveborn
	Z3750	Multiple births, unspecified, all liveborn
	Z3751	Triplets, all liveborn
	Z3752	Quadruplets, all liveborn
	Z3753	Quintuplets, all liveborn
	Z3754	Sextuplets, all liveborn
	Z372	Twins, both liveborn
	Z3759	Other multiple births, all liveborn
	Z376	Other multiple births, some liveborn
	Z3760	Multiple births, unspecified, some liveborn
	Z3761	Triplets, some liveborn
	Z3762	Quadruplets, some liveborn
	Z3763	Quintuplets, some liveborn
	Z3764	Sextuplets, some liveborn
	Z3769	Other multiple births, some liveborn
	Z379	Outcome of delivery, unspecified
	Z373	Twins, one liveborn and one stillborn
	V270	Outcome of delivery, single liveborn
	V275	Outcome of delivery, other multiple birth, all liveborn
	V272	Outcome of delivery, twins, both liveborn
	V276	Outcome of delivery, other multiple birth, some liveborn
	V273	Outcome of delivery, twins, one liveborn and one stillborn
	650	Normal delivery
	V279	Outcome of delivery, unspecified outcome of delivery
	O80	Encounter for full-term uncomplicated delivery
	O82	Encounter for cesarean delivery without indication

Appendix Table 2. CPT Codes for Delivery

	CPT Codes	Code Description
Delivery	59614<br><br><br>59618<br>59620<br><br><br>59622<br><br><br>59400<br>59409<br>59410<br>59510<br>59514<br>59515<br>59610<br>59612	Vaginal delivery only, after previous cesarean delivery (with or without episiotomy and/or forceps)<br>Delivery Procedures After Previous Cesarean Delivery<br>Cesarean delivery only, following attempted vaginal delivery after previous cesarean delivery<br><br>Cesarean delivery only, following attempted vaginal delivery after previous cesarean delivery<br><br>Vaginal Delivery, Antepartum and Postpartum Care Procedures<br>Vaginal delivery only (with or without episiotomy and/or forceps)<br>Vaginal delivery only (with or without episiotomy and/or forceps)<br>Cesarean Delivery Procedures<br>Cesarean delivery only<br>Cesarean delivery only<br>Delivery Procedures After Previous Cesarean Delivery<br>Vaginal delivery only, after previous cesarean delivery (with or without episiotomy and/or forceps)

Appendix Table 3. CPT Codes for Hepatitis-B Screening

	CPT Codes	Code Description
HBsAg	80055	Obstetric Panel (AMA)
	87340	Hepatitis-B Surface Antigen, Reflex Confirmation
	87341	Hepatitis-B Surface Antigen, Confirmation
	80074	Hepatitis Panel, Acute (AMA)
	80081	Obstetric Panel (AMA) with HIV

Appendix Table 4. ICD 9/10 Codes for Chronic Hepatitis B

	ICD 9/10 Codes	Code Description
CHB	B18.1<br>B18.0<br>B19.10<br>Z22.51<br>V02.61<br>070.32<br><br>070.33<br><br>070.22<br>070.2	CHB without HDV<br>CHB with HDV<br>Unspecified viral hepatitis B without hepatic coma<br>Carrier of viral hepatitis B<br>Carrier of viral hepatitis B<br>CHB without mention of hepatic coma or hepatitis delta<br><br>CHB without mention of hepatic coma with hepatitis delta<br><br>CHB with hepatic coma without hepatitis delta<br>CHB with hepatic coma with hepatitis delta

Appendix Table 5. CPT Codes for CHB Monitoring

	CPT Codes	Code Description
Alanine Aminotransferase (ALT)	80076<br>84460<br>80053	Hepatic function panel<br>Alanine Aminotransferase (ALT)<br>Comprehensive metabolic panel (CMP)
HBV DNA Test	87515<br>87516<br>87517	Hepatitis B virus detection by nucleic acid using direct probe technique<br>Hepatitis B virus DNA, Qualitative, Real-Time PCR<br>Hepatitis B Virus DNA, Qualitative Real-Time PCR
HBeAg Test	87350	Hepatitis B e Antigen

Appendix Table 6. Antiviral Treatment Codes

HBV antivirals	Brand and generic names	Tenofovir/tenofovir disoproxil fumarate/Viread/ Tenofovir alafenamide/Vemlidy<br>Entecavir/ Baraclude<br>Lamivudine/Epivir-HBV/Epivir<br>Adefovir/Hepsera<br>telbivudine/Tyzeka<br>Emtricitabine/ Emtriva<br>Pegylated interferon alpha-2a/Pegasys/Pegasys-P<br>Interferon alfa-2b/Intron A<br>Pegylated interferon alpha-2b/Peg-Intron<br>HIV combination drugs that contains lamivudine or tenofovir (Atripla, Biktarvy, Cimduo, Combivir, Complera, Delstrigo, Descovy, Epzicom, Genvoya, Odefsey, Stribild, Symfi, Symfi Lo, Symtuza, Triumeq, Trizivir, Truvada, Dovato)

Appendix Table 7. Demographics characteristics of 506,794 unique pregnancies between 1/1/2015 and 12/31/2019 in continuous enrollment at least 42 weeks before delivery

Characteristics	Categories	N	(%)
**Age (yrs) at the time of pregnancy**	**15-19**	6,519	1.3%
	**20-29**	169,590	33.5%
	**30-39**	304,651	60.1%
	**40-55**	26,034	5.1%
**Race**	**Asian**	42,625	8.4%
	**Black**	47,354	9.3%
	**Hispanic**	73,769	14.6%
	**White**	333,792	65.9%
	**Unknown**	9,254	1.8%
**Education**	**Less than 12th grade**	2,001	0.4%
	**High school diploma**	93,469	18.4%
	**Less than bachelor's degree**	272,849	53.8%
	**Bachelor's degree or higher**	137,362	27.1%
	**Unknown**	1,113	0.2%
**Number of children**	**0**	56,762	11.2%
	**1**	165,627	32.7%
	**2**	174,308	34.4%
	**>=3**	110,097	21.7%

Appendix Table 8. Demographic characteristics of 1,437 HBsAg positive pregnancies, 2015-2019

	Categories	N	%
**Age (yrs) at the time of pregnancy**	**15-19**	3	0.2%
	**20-29**	232	16.1%
	**30-39**	1,026	71.4%
	**40-55**	176	12.3%
**Race**	**Asian**	661	46.0%
	**White**	418	29.1%
	**Black**	185	12.9%
	**Hispanic**	130	9.1%
	**Unknown**	43	3.0%
**Education**	**Less than bachelor's**	680	47.3%
	**Bachelor's degree plus**	505	35.1%
	**High school diploma**	242	16.8%
	**Less than 12th grade**	5	0.4%
	**Unknown**	5	0.4%
**Number of children**	**0**	104	7.2%
	**1**	476	33.1%
	**2**	574	39.9%
	**>=3**	283	19.7%

Appendix Table 9. Monitoring of CHB with testing for ALT, HBV DNA and HBeAg among 1,437 unique pregnancies diagnosed with CHB, 2015-2019

	2015	2016	2017	2018	2019	TOTAL
**CHB MONITORING DURING PREGNANCY**						
**Having at least one CPT code for ALT during pregnancy**	186<br>59.7%	197<br>67.2%*	210<br>70.4%**	184<br>70.0%**	192<br>70.8%**	969<br>67.4%
**Having at least one CPT code for HBV DNA during pregnancy**	114<br>35.5%	135<br>46.1%*	154<br>51.9%**	107<br>40.7%	126<br>46.5%*	637<br>44.3%
**Having at least one CPT code for HBeAg during pregnancy**	89<br>28.5%	102<br>34.8%	100<br>33.3%	74<br>28.1%	90<br>33.2%	455<br>31.6%
**Having at least one CPT code for ALT in conjunction with either one code for HBeAg or HBV DNA during pregnancy**	126<br>40.3%	138<br>47.1%	157<br>52.5%	112<br>42.6%	126<br>46.5%	659<br>45.8%
**CHB MONITORING IN THE 12 MONTHS AFTER DELIVERY**						
**Having at least one CPT code for ALT within 12 months after delivery**	101<br>45.5%	96<br>48.5%	100<br>49.3%	79<br>44.4%	76<br>46.6%	452<br>47.0%
**Having at least one CPT code for HBV DNA within 12 months after delivery**	58<br>26.1%	62<br>31.6%	68<br>33.5%	45<br>25.3%	42<br>25.8%	275<br>28.6%
**Having at least one CPT code for HBeAg within 12 months after delivery**	32<br>14.4%	23<br>11.7%	27<br>13.3%	21<br>11.8%	19<br>11.7%	122<br>12.7%
**Having at least one CPT code for ALT in conjunction with either one code for HBeAg or HBV DNA within 12 months after delivery**	53<br>23.8%	58<br>29.6%	58<br>28.57%	43<br>24.2%	39<br>23.9%	251<br>26.1%

*** p<0.05 compared with 2015, **p<0.01 compared with 2015.**

Appendix Table 10. Demographics factors associated with HBV-directed monitoring (ALT + HBV DNA/HBeAg) 0-12 months after delivery among 962 unique pregnancies with CHB diagnosis in continuous enrollment at least 12 months after delivery, 2015-2020

**Characteristics**		**Number (%)**	**Univariate analysis**		**Multivariate analysis**	
			**Odds Ratio, (95% CI)**	**P**	**Adjusted Odds Ratio, (95% CI)**	**p**
**Age**	15-29	**27 (18.9** %)	(ref)		(ref)	
	30-39	192 (27.1%)	1.60 (1.01-2.51)	**0.04**	1.38 (0.86-2.23)	0.19
	40-55	32 (28.8%)	1.74 (0.96-3.12)	0.06	1.46 (0.79-2.72)	0.23
**Race**	Asian	164 (36.4%)	(ref)		(ref)	
	Black	28 (22.9%)	0.52 (0.33-0.83)	**<0.01***	0.63 (0.38-1.02)	0.06
	Hispanic	12 (15.6%)	0.32 (0.17-0.62)	**<0.01***	0.34 (0.17-0.65)	**<0.01***
	White	42 (14.9%)	0.31 (0.21-0.45)	**<0.01***	0.30 (021-0.45)	**<0.01***
**Education**	<= high school	31 (19.9%)	(ref)		(ref)	
	Less than bachelor's	119 (26.5%)	1.45 (0.93-2.27)	0.10	1.33 (0.84-2.11)	0.23
	Bachelor's degree plus	99 (28.1%)	1.57 (1.00-2.48)	0.05	1.24 (0.37-2.02	0.37
**Number of children**	0	7 (11.1%)	(ref)		(ref)	
	1	80 (28.8%)	3.23 (1.41-6.43)	**<0.01***	3.10 (1.25-7.71)	**0.02**
	2	108 (26.2%)	2.84 (1.26-6.43)	**0.01**	2.45 (1.00-6.07)	0.05
	>=3	56 (26.8%)	2.93 (1.26-6.80)	**0.01**	2.95 (1.17-7.44)	**0.02**

Note: Boldface indicates statistical significance (p<0.05, *p<0.01)

Appendix Table 11. Correlation analysis of demographics on HBV antiviral treatment during pregnancy among 1,437 unique pregnancies, 2015-2019

**Characteristics**		**Number (%)**	**Univariate analysis**	
			**OR, (95% CI)**	**p**
**Age (yrs)**	**15-29**	15 (6.4%)	(ref)	
	**30-39**	76 (7.4%)	1.17 (0.66-2.08)	0.58
	**40-55**	10 (5.7%)	0.88 (0.39-2.02)	0.77
**Race**	**Asian**	70 (10.6%)	(ref)	
	**Black**	6 (3.2%)	0.28 (0.12-0.66)	**<0.01***
	**Hispanic**	3 (2.3%)	0.20 (0.06-0.64)	**<0.01***
	**White**	18 (4.3%)	0.38 (0.22-0.65)	**<0.01***
**Education**	**high school or less**	19 7.7%	(ref)	
	**Less than bachelor's**	43 (6.3%)	0.81 (0.46-1.42)	0.46
	**Bachelor's degree plus**	38 (7.5%)	0.98 (0.55-1.73)	0.94
**Number of children**	**0**	8 (7.7%)	(ref)	
	**1**	33 (6.9%)	0.89 (0.40-1.99)	0.78
	**2**	46 (8.0%)	1.04 (0.487-2.28)	0.91
	**>=3**	14 (4.95%)	0.62 (0.25-1.54)	0.31

Note: Boldface indicates statistical significance (*p<0.01)

Appendix Table 12. Demographics factors associated with claims for HBV antiviral treatment 0-12 months after delivery among the pregnancies with CHB diagnosis in continuous enrollment at least 12 months after delivery, 2015-2020

**Characteristics**		**Number (%)**	**Univariate analysis**	
			**Odds Ratio, (95% CI)**	**P**
**Age (yrs)**	15-29	7 (4.9%)		
	30-39	47 (6.6%)	1.38 (0.61-3.12)	0.44
	40-55	6 (5.4%)	1.11 (0.36-3.40)	0.86
**Race†**	Asian	40 (8.9%)		
	Black	4 (3.3%)	0.35 (0.12-0.99)	0.05
	Hispanic	2 (2.6%)	0.27 (0.06-1.16)	0.08
	White	13 (4.6%)	0.50 (0.26-0.94)	**0.03**
**Education†**	<= high school	11 (7.1%)		
	Less than bachelor's	25 (5.6%)	0.78 (0.37-1.61)	0.51
	Bachelor's degree plus	23 (6.5%)	0.92 (0.44-1.93)	0.82
**Number of children**	0	2 (3.2%)		
	1	21 (7.6%)	2.49 (0.56-10.91)	0.22
	2	30 7.3%)	2.40 (0.55-10.28)	0.24
	>=3	7 (3.4%)	1.06 (0.21-5.22)	0.07

Note: Boldface indicates statistical significance ($p<0.05$)
† Unknown in one woman

Appendix Figure 1. Flow chart cascade of hepatitis B testing, care, and treatment among pregnancies in Optum, 2015-2020.

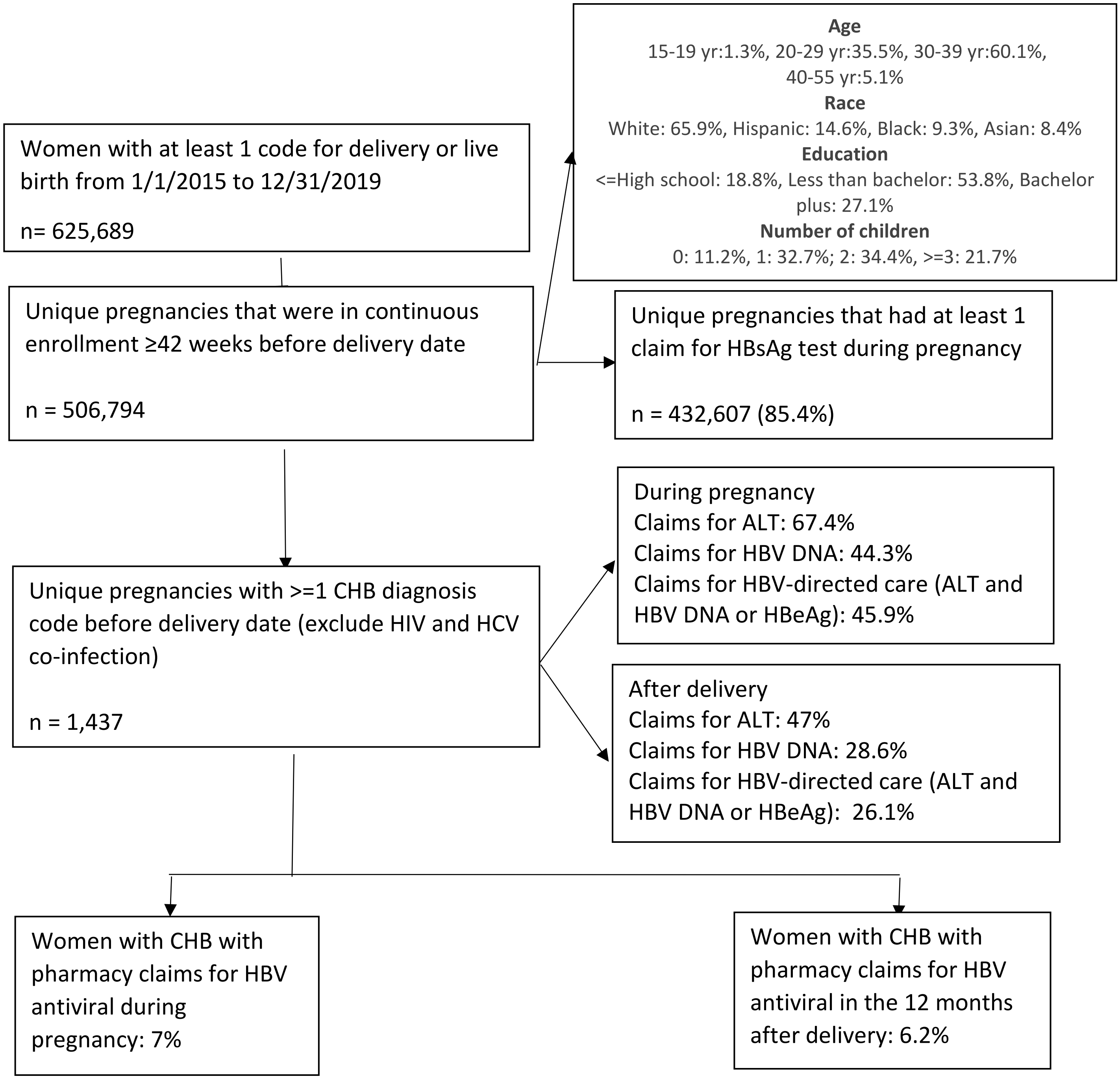

Appendix Figure 2. HBV disease monitoring in the 12 months after delivery, 2015-2020

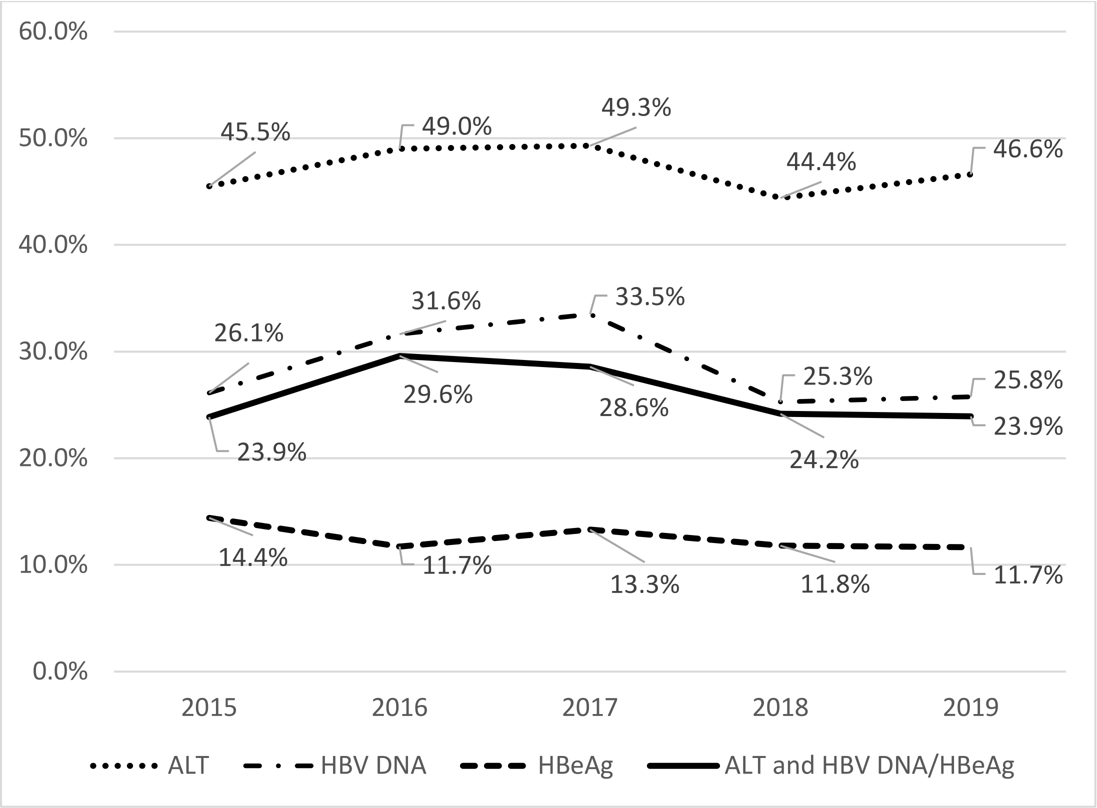

Supplement: Appendix [file NIHMS1977675-supplement-Appendix.pdf]
